# Comparison of Electrocardiographic Parameters by Gender in Heart Failure Patients with Preserved Ejection Fraction via Artificial Intelligence

**DOI:** 10.3390/diagnostics13203221

**Published:** 2023-10-16

**Authors:** Rustem Yilmaz, Ersoy Öz

**Affiliations:** 1 Department of Cardiology, Faculty of Medicine, Samsun University, Samsun 33805, Turkey; 2 Department of Statistics, Yildiz Technical University, Istanbul 34220, Turkey; ersoyoz@yildiz.edu.tr

**Keywords:** heart failure with preserved ejection fraction, artificial intelligence, gender-specific electrocardiographic parameters

## Abstract

Background: Heart failure (HF) causes high morbidity and mortality worldwide. The prevalence of HF with preserved ejection fraction (HFpEF) is increasing compared with HF with reduced ejection fraction (HFrEF). Patients with HFpEF are a patient group with a high rate of hospitalization despite medical treatment. Early diagnosis is very important in this group of patients, and early treatment can improve their prognosis. Although electrocardiographic (ECG) findings have been adequately studied in patients with HFrEF, there are not enough studies on these parameters in patients with HFpEF. There are very few studies in the literature, especially on gender-specific changes. The current research aims to compare gender-specific ECG parameters in patients with HFpEF based on the implications of artificial intelligence (AI). Methods: A total of 118 patients participated in the study, of which 66 (56%) were women with HFpEF and 52 (44%) were men with HFpEF. Demographic, echocardiographic, and electrocardiographic characteristics of the patients were analyzed to compare gender-specific ECG parameters in patients with HFpEF. The AI approach combined with machine learning approaches (gradient boosting machine, k-nearest neighbors, logistic regression, random forest, and support vector machines) was applied for distinguishing male patients with HFpEF from female patients with HFpEF. Results: After determining the parameters (demographic, echocardiographic, and electrocardiographic) to distinguish male patients with HFpEF from female patients with HFpEF, machine learning methods were applied, and among these methods, the random forest model achieved an average accuracy of 84.7%. The random forest algorithm results showed that smoking, P-wave dispersion, P-wave amplitude, T-end P/(PQ*Age), Cornell product, and P-wave duration were the most influential parameters for distinguishing male patients with HFpEF from female patients with HFpEF. Conclusions: The proposed model serves as a valuable tool for physicians, facilitating the diagnosis, treatment, and follow-up for distinguishing male patients with HFpEF from female patients with HFpEF. Analyzing readily accessible electrocardiographic parameters empowers medical professionals to make informed decisions and provide enhanced care to a wide range of individuals.

## 1. Introduction

Heart failure (HF) causes high morbidity and mortality worldwide. Especially in developed and developing countries, the number of patients with HF is rapidly increasing due to longer life expectancy, and the major causes of this situation are increasing chronic ischemic heart disease and hypertension [1]. The prevalence of HF with preserved ejection fraction (HFpEF) is increasing compared with HF with reduced ejection fraction (HFrEF) and affects more women than men in a 2:1 ratio [2,3]. Patients with HFpEF are a patient group with a high rate of hospitalization despite medical treatment. Although their treatment is similar to patients with HFrEF, their prognosis is poor. Early diagnosis is very important in this group of patients, and early treatment can improve their prognosis [4].

HFpEF is a clinical syndrome; it is defined in individuals who have signs and symptoms of HF, evidence of structural and/or functional cardiac abnormalities and/or elevated natriuretic peptides (NPs), and an LVEF > of 50% [5]. Left ventricular diastolic dysfunction (LVDD) is considered a precursor to HFpEF. LVDD usually manifests as increased atrial volume and high filling pressure accompanied by LV mass, abnormal relaxation, and decreased LV compliance [6,7]. It is accompanied by some electrocardiographic (ECG) changes for HFrEF. These include prolonged PR intervals, low voltages, QRS prolongations, and QT prolongations, distribution, and variability [8,9,10,11]. In addition, several ECG features have been shown to be very helpful in identifying HFrEF in primary care [12,13]. Similarly, ECG features may help in the selection of patients requiring echocardiography for HFpEF. However, the ECG features associated with HFpEF are less well known. A recent meta-analysis found a higher incidence of right bundle branch block and atrial fibrillation in HFpEF compared with HFrEF [14]. Although electrocardiographic (ECG) findings have been adequately studied in patients with HFrEF, there are not enough studies on these parameters for patients with HFpEF. Currently, there is a significant gap in the current literature regarding these gender-specific ECG parameters. Moreover, there are no studies that have gender-specifically investigated ECG parameters to distinguish male patients with HFpEF from female patients with HFpEF using an AI-based clinical approach.

AI plays a crucial role in numerous clinical decision support systems, facilitating the use of computational methods to make inferences that are comparable to human reasoning processes [15]. The strategies presented in this context are founded upon medical information that has been either explicitly encoded or automatically generated from medical data using machine learning techniques. AI has the potential to facilitate the prioritization of patients’ wellbeing and enable them to make independent and well-informed choices regarding their healthcare in conjunction with medical professionals [16]. In the realm of cardiology, AI has provided more accurate and rapid diagnostic methods in fields such as the analysis of electrocardiography data, identification of arrhythmias, and assessment of cardiovascular risk factors. By aiding cardiologists in processing large datasets and identifying complex patterns, AI simplifies the early diagnosis of heart diseases and the creation of personalized treatment plans. The incorporation of AI in cardiology allows for improved outcomes in managing severe cardiovascular issues like coronary artery disease, heart failure, and arrhythmias, offering the potential to safeguard patient health and extend their lifespans. Building on the valuable information emphasized in various medical studies, the aim of this study is to determine gender-specific ECG parameters that distinguish male and female patients with HFpEF based on the implications of AI. For achieving this aim, first, descriptive statistics are obtained on the dataset used in the study. Then, evaluations are made with the traditional statistical analysis methods. In the application part of this study, analyses are mainly performed with AI algorithms. For the purpose of this study, classification algorithms (gradient boosting machine, k-nearest neighbors, logistic regression, random forest, and support vector machines) are used to distinguish patients with HFpEF by gender in the dataset. For the classification algorithms, the most suitable parameters for each algorithm are determined by parameter optimization. Then, attribute importance levels for the most successful classification algorithm are investigated and comments are made about it.

## 2. Materials and Methods

### 2.1. Study Design and Data

The present study was an observational study. A total of 118 patients, 52 men and 66 women, with HFpEF who presented to the Samsun University, Samsun Training and Research Hospital cardiology department between November 2022 and August 2023 were included (please see Supplementary Table S1 for the whole dataset). HFpEF was defined according to the European Society of Cardiology guidelines (ESC) for the diagnosis and management of acute and chronic heart failure as patients presenting with signs and symptoms of HF (e.g., dyspnea, paroxysmal nocturnal dyspnea (PND), orthopnea, ankle edema, or distension of the jugular veins), evidence of structural and/or functional cardiac abnormalities, and/or elevated natriuretic peptides (NPs) and a normal or near-normal left ventricular ejection fraction (LVEF > 50%), evidence of cardiac dysfunction by echocardiography (e.g., abnormal left ventricular filling and elevated filling pressures) [5]. The patients who consented to participate in this study were over 18 years of age and were diagnosed with HFpEF.

Those who refused to participate in this study and/or had severe coronary artery disease, moderate to severe valvular heart disease, chronic obstructive pulmonary disease, malignant disease, atrial fibrillation (AF), left bundle branch block (LBBB), second- and third-degree atrioventricular block (A-V), advanced renal and liver failure, drug toxicity, electrolyte imbalance, and hyperthyroidism were excluded. This study was conducted in accordance with the Declaration of Helsinki and approved by the Clinical Research Ethics Committee of Samsun University (protocol codes 2022/11/10 and 09.11.2022). The information was obtained from the patients or their first-degree relatives. The aim of the meeting was to obtain the following information:

(i) Age, gender, height, weight, smoking, drug use, arterial hypertension, diabetes mellitus, coronary artery disease, and history of thyrotoxicosis were asked to determine the demographic and clinical characteristics of the patients.

(ii) Twelve-lead surface ECG recordings were obtained from all patients using a 12-lead electrocardiography machine with a speed of 25 mm/s and a calibration of 10 mm/mV to study P-wave variability, its distribution and amplitude, QRS duration, QT and QTc duration, PQ distance, PR distance, T-end Q, T-end P interval, ventricular repolarization, and depolarization ECG parameters. 

(iii) In accordance with the most recent American Society of Echocardiography and European Association of Cardiovascular Imaging guidelines for quantification of the ventricles using echocardiography in adults, two-dimensional M-mode and Doppler echocardiography was performed on all patients using the General Electric Vivid 7 echocardiography machine to determine left ventricular ejection fraction (EF), left atrial diameter, left ventricular systolic and diastolic functions, diastolic diameter, and valve pathology [17].

Definitions of ECG parameters: 

P-wave dispersion; difference between the longest and shortest P-wave duration recorded from multiple ECG leads, P-wave amplitude; peak of P-wave to the isoelectric line of TP interval in lead D2, P-wave duration; beginning of P-wave until end of P-wave, Cornell product; (RaVL + SV3)*ORS duration, PQ and PR interval; beginning of P-wave until onset of Q- or R-wave, QT interval; interval between Q-wave onset and end of T-wave, QTc interval: QT interval is often corrected for heart rate (QTc) by Bazett’s formula, T-end Q; end of T-wave to Q-wave onset, T-end P interval; end of T-wave to P-wave onset and QRS duration; beginning of Q-wave until end of R-wave.

Depending on the above explanations, statistical analyses and AI algorithms were performed with 26 variables (name used in the field of AI: features) of 118 patients. There were no missing data in the dataset in this study. Classification was performed using the AI algorithms described in Section 2.3, and the target variable for these classifications was gender (female patients with HFpEF and male patients with HFpEF).

### 2.2. Statistical Analysis

The independent samples t-test is used to determine whether there is a statistically significant difference between the means of two independent groups. This test assumes that the group variances are equal, and the group distributions are normal. When at least one of these assumptions is not met, the Mann–Whitney U test can be used as an alternative. Correlation analysis was performed to see the relationships between variables. Pearson correlation was utilized if the distribution of the variables met the normal distribution; otherwise, Spearman’s rho correlation was used. Additionally, the chi-squared test was employed to examine whether there was a dependency between categorical variables. Where the p-value is significant, the level of significance is indicated by * or **. * and ** indicate that the control is at the 95% and 99% confidence level, respectively.

### 2.3. Artificial Intelligence

In this study, AI algorithms gradient boosting machine (GBM), k-nearest neighbors (kNN), logistic regression (LR), random forest (RF), and support vector machines (SVMs) were used. Brief descriptions of these algorithms and other methods are provided below:

Gradient boosting machines (GBMs): GBMs are a family of powerful machine learning techniques that have shown considerable success in a wide range of practical applications. The learning procedure consecutively fits to the new models to provide a more accurate estimate of the response variable. The principal idea behind this algorithm is to construct the new base learners to be maximally correlated with the negative gradient of the loss function, associated with the whole ensemble [18]. It should be noted that the essence of a GBM lies in its nature as an ensemble of weak learners, typically in the form of decision trees. This ensemble approach allows for the GBM to build a strong predictive model by combining the predictions of these individual weak learners iteratively. In essence, a GBM aims to correct the errors made by the previous base learners in each iteration, thereby continuously improving the overall model’s accuracy. This iterative, ensemble-based learning is a key feature that sets GBMs apart from other machine learning techniques.

k-nearest neighbors (kNN): The kNN algorithm is a type of machine learning algorithm used for classification and regression. It works by finding the k closest labeled examples in the training dataset to an unlabeled example and assigning the label of the majority of those k examples to the unlabeled example. The key concept of kNN is easy to understand and can be used for a variety of applications [19]. 

Logistic regression (LR): LR is a widely used algorithm in classification problems. It is particularly suitable for binary classification (e.g., sick / healthy). LR is useful for understanding prediction results and which features of the model have more influence on the prediction.

Random forest (RF): RF is applicable to both classification and regression tasks. It belongs to the ensemble learning methods category and operates by amalgamating the outcomes of multiple decision trees. In addition to combining the results of decision trees, RF utilizes diverse subsets of data and their corresponding features. This leads to the creation of a multitude of decision trees, each contributing to a distinct prediction of the problem. Subsequently, the outcomes of these individual trees are aggregated by the RF. In classification scenarios, the ultimate verdict is determined through a voting mechanism. The class assignment of input samples is established by the majority vote from the decision trees [20].

Support vector machine (SVM): An SVM is based on the principle of finding the hyperplane between the classes to be predicted. It is an algorithm used in classification and regression problems. It is particularly effective in high-dimensional datasets to identify complex boundaries between data and to separate classes. It can be adapted to different data types using different kernel functions.

k-fold cross-validation: k-fold cross-validation is a method used to evaluate the performance of a model created by an AI algorithm. The dataset is divided into k parts, then trained on k-1 parts and validated on 1 part. This process is repeated k times, and each part is used for both training and validation. The results are averaged to evaluate the overall performance of the model.

Feature importance: A value that measures how many different input features in the dataset upon which a machine learning model is trained contribute to the model’s predictions. These values help to understand which features have more influence on the prediction results and are used to improve feature selection or data understanding.

### 2.4. Performance Evaluation

In the fields of machine learning and statistics, accuracy, recall, precision, Cohen’s Kappa score, and the *F1* score are often-used performance metrics, particularly for assessing the performance of classification models [21]. A classification model’s performance is assessed using the confusion matrix metric. This matrix shows the relationship between the true class and the predicted classes. “True Positive (TP)”, “False Positive (FP)”, “True Negative (TN)”, and “False Negative (FN)” are the four key terms that make up the confusion matrix. These terms indicate how accurately or inaccurately the model predicts the class.

Accuracy: Accuracy is the classification model’s correct predictions ratio to the total number of samples. In general, this metric is employed to evaluate the model’s general performance. Accuracy is calculated with Equation (1):

(1) $$Accuracy=\frac{TP+TN}{TP+TN+FP+FN}$$

Precision: Precision, commonly referred to as positive predictive value, assesses how accurately a model makes positive predictions. Precision is calculated with Equation (2):

(2) $$Precision=\frac{TP}{TP+FP}$$

The model’s ability to avoid false positives is evidence of a high precision. 

Recall: Often referred to as sensitivity or true positive rate, recall refers to a model’s ability to capture every positive instance in the dataset. 

Recall is calculated with Equation (3):

(3) $$Recall=\frac{TP}{TP+FN}$$

A high recall demonstrates the model’s capacity to precisely identify every relevant positive instance.

*F1* Score: Precision and recall’s harmonic mean makes up the *F1* score. A better balance between recall and precision is indicated by a higher *F1* score, which runs from zero to one.

*F1* score is calculated as in Equation (4):

(4) $$F1\;Score=\frac{2\times Precision\times Recall}{Precision+Recall}$$

In summary, accuracy assesses overall correctness, precision assesses positive predictions’ accuracy, recall assesses the ability to identify every positive instance, and the *F1* score, the combination of recall and precision, provides a balanced evaluation of a classification model’s performance. These metrics are frequently utilized to evaluate the quality of the predictions made by a model.

Cohen’s kappa score: Cohen’s kappa (κ) score is a statistical measure that assesses how much agreement there is between two different observers or a model’s classification results [22]. The kappa score indicates how far the classification results are from randomness and measures the agreement between observers or models. Equation (5) is used to determine the kappa score:

(5) $$\kappa =\frac{Po-Pe}{1-Pe}$$

Here, P_o_ represents the agreement of classifications performed by observers or the model. In other words, it is the percentage of observations where observers or the model is assigned the same class label. P_e_ represents the probability of two observers or the model predicting the same class in a random classification scenario. This represents the situation where each observer or the model assigns class labels randomly [23]. A Cohen’s kappa score of +1 denotes perfect agreement between the two observers, while a score of −1 denotes perfect disagreement. In other words, if a classification model’s Cohen’s kappa score approaches +1, it means that the model’s classification predictions are very close to the actual values and significantly distant from randomness. This situation indicates that the model’s results are reliable and consistent.

Receiver operating characteristic: Receiver operating characteristic (ROC) analysis is a method for evaluating the performance of binary classification models [24]. In ROC analysis, the performance of a model can be evaluated by examining the ROC curve’s shape and considering the area under the curve (AUC). A higher AUC indicates a better-performing model. The ROC curve can be constructed using either the predictions of the classification model or the resulting probability estimates for each class. The AUC created using these methods may be different because these two methods represent different approaches and therefore produce different results.

### 2.5. Artificial Intelligence Application Procedure

The steps of the application carried out with AI algorithms are provided below:

1. Preparation of the dataset: Outlier analysis was performed on a feature basis in the dataset. Frequency distribution was examined for the target variable, a group (female patients with HFpEF and male patients with HFpEF).
2. Splitting the dataset: For classification purposes, before running the AI algorithms, the dataset was randomly divided into 80% training and 20% testing.
3. Running AI algorithms: The training set was used both to determine the optimum parameters of AI algorithms and to establish the models. Optimum parameters were obtained for each algorithm using the grid search method with k-fold cross-validation, where *k* is equal to 5. Final models were established with these optimum parameters. Then, the models were tested with the test set and performance evaluation metrics were obtained.

In dividing the dataset, the second and third steps were repeated 50 times to reduce the effects of randomness and to obtain more reliable results.

4. Obtaining the average classification results: In each process that was repeated 50 times, performance metric values were obtained from every dataset for each algorithm, and average performance metric values were obtained using these values.
5. Feature importance for the algorithm with the highest average accuracy value and visualization of the results: Feature importance scores were determined for the algorithm with the highest average classification performance. In other words, when determining feature importance scores, the appropriate feature selection method was used according to the algorithm with the highest average classification performance. Feature importance scores present the relative importance levels of features effective in distinguishing the target variable, that is, group (female or male), for patients with HFpEF.

All analyses in this study were carried out with the Python programming language, which is an open-source project. Various submodules of the “scikit-learn” module, the “numpy”, “pandas”, “matplotlib”, and “statsmodels” submodules, were used to perform specific tasks such as data analysis, visualization, model building, and evaluation operations.

## 3. Results

A total of 118 people participated in this study, of which 66 (56%) were female patients with HFpEF and 52 (44%) were male patients with HFpEF. The descriptive statistics of the variables (features) of the patients whose data were used within the scope of this study are provided in Table 1 as minimum, maximum, median, mean, standard deviation, and percentage.

**Figure 1:**
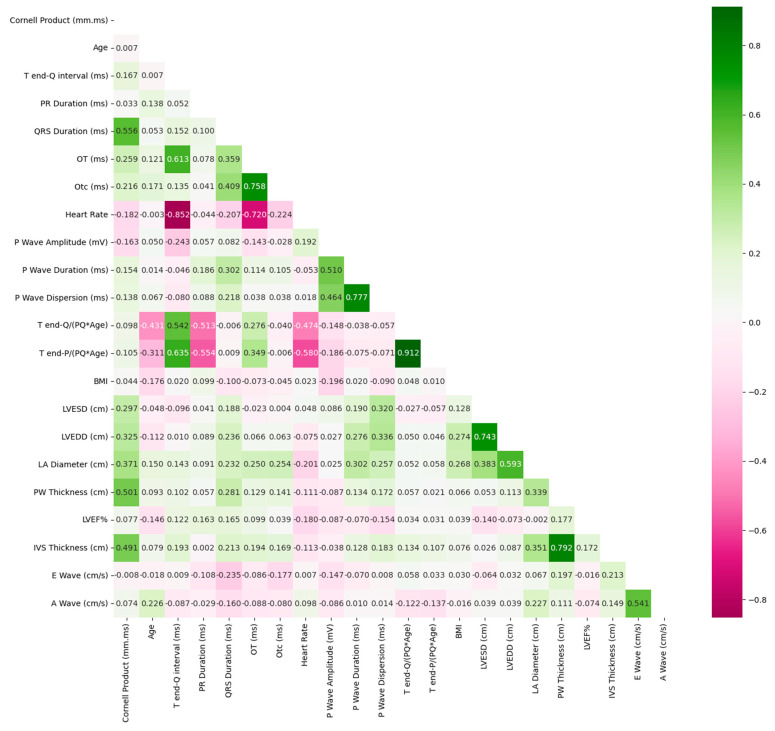
Correlation matrix.

In Figure 1, Spearman’s rho coefficients of variables can be seen. If the relationship between them is positive, the related cell is colored in green. Otherwise, the cell is colored in red. The highest positive relationship is between T-end P/(PQ*Age) and T-end Q/(PQ*Age). The highest negative relationship is between heartrate and T-end Q interval (ms). According to these results, while T-end P/(PQ*Age) increases, T-end Q/(PQ*Age) also increases, but while heartrate increases, T-end Q interval (ms) decreases.

Upon examining Table 2, for males and females, it was concluded that there is no statistical difference between the mean of Cornell product, age, T-end Q interval, PR duration, OT, Otc, heartrate, T-end Q/(PQ*Age), T-end P/(PQ*Age), BMI, LVESD, LVEDD, LA diameter, PW thickness, LVEF%, IVS thickness, and A-wave. However, there is a statistically significant difference between men and women at the 95% confidence level, in terms of means of QRS duration and E-wave, and at 99% confidence level, in terms of means of P-wave amplitude, P-wave duration, and P-wave dispersion. Although the means of QRS duration, P-wave amplitude, P-wave duration, and P-wave dispersion for women are lower than for men, the mean of E-wave is higher than for men. 

Chi-square test results (Table 3) showed that while there is no significant relationship between men and women in terms of DM, it can be said that there is a significant relationship at the 99% confidence level for smoking (*p*-value < 0.001) and 95% confidence level for HT (*p*-value = 0.02). While 13.6% of women smoke, 63.5% of men smoke. The number of women with hypertension among themselves is ten times that of those without hypertension. In men, the number of those with hypertension among themselves is three times that of those without hypertension.

The averages of the classification results obtained as a result of the AI algorithms are provided in Table 3. This table shows the performance evaluation metrics with 95% confidence intervals (CI) for the classification algorithms. In Table 3, the RF algorithm achieves the most successful classification based on accuracy and other performance evaluation metrics. The other performance evaluation metrics of the RF algorithm such as precision, recall, *F1* score, and AUC are also compatible with the accuracy value. A similar evaluation can be made for other algorithms and metrics. 

Another measure that shows the success (suitability) of the models is the kappa score. When Table 3 is examined, it is seen that the lowest kappa value was calculated for the kNN algorithm, and the highest kappa value was calculated for the RF algorithm. The kNN algorithm’s kappa value is almost 0, indicating that the algorithm performs almost the same as random guesses. 

**Figure 2:**
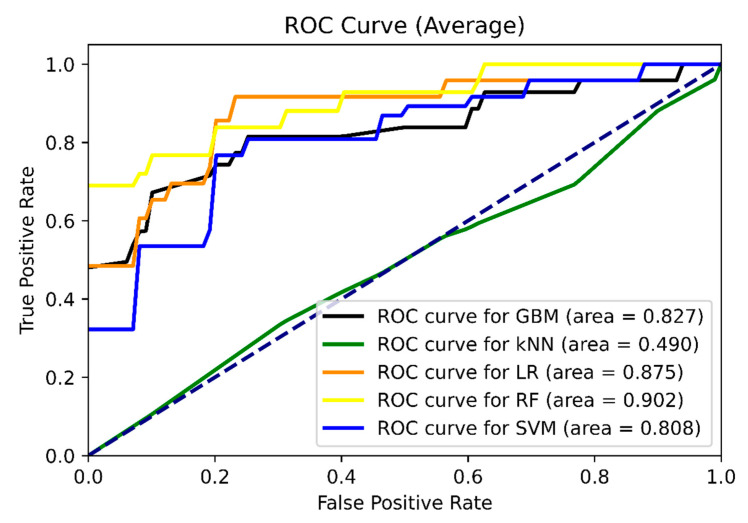
Average ROC curves for classification algorithms.

According to performance evaluation metrics, GBM was the most successful algorithm after the RF algorithm. LR and SVM are the most successful algorithms after GBM, and their performance evaluation metrics are very close to each other. Additionally, average AUC values of the classification algorithms are shown in Figure 2 to provide a visual evaluation with performance evaluation metrics. The (average) ROC curves here are plotted using the probability estimates obtained for each class. 

**Figure 3:**
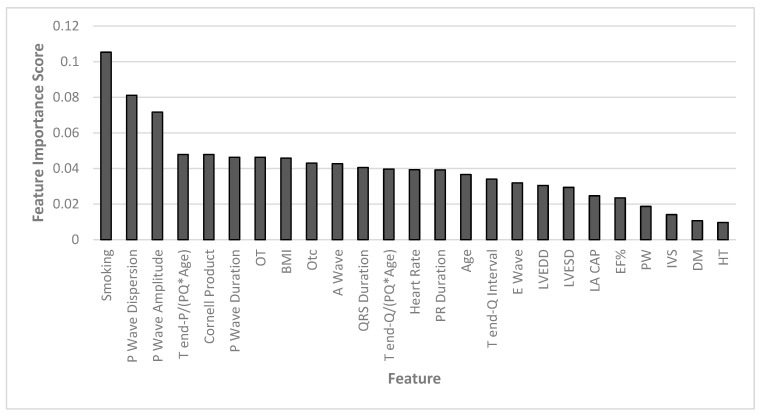
Feature importance.

RF stands out as the most effective algorithm for classifying both female and male patients in our study. To gain deeper insight into the significance of input features in the classification process, we employed a feature importance function specifically designed to work seamlessly with ensemble algorithms like RF. This function plays a pivotal role in assessing the extent to which each input feature contributes to the model’s performance and how it impacts prediction outcomes. The feature importance function quantifies the relevance of each feature by assigning a value between 0 and 1. These values represent the contribution of each feature to the model’s decision-making process. Typically, these importance values are normalized to ensure that their sum equals 1. This normalization facilitates a more intuitive understanding of the relative importance of each feature within the model. In Figure 3, we present the scores obtained from our calculations, which shed light on the features’ influence on gender classification. These scores not only highlight which features are instrumental in the classification process but also provide valuable insights into the hierarchical importance of these features. The RF algorithm offers two ways to compute feature importance [25,26]: 1. Gini importance is calculated from the RF’s structure, where each decision tree selects features based on criteria like Gini impurity or information gain for classification tasks and variance reduction for regression. The feature importance is measured by how much it decreases impurity during splits, with the average importance across all trees in the forest serving as the final measure [27]. 2. Mean decrease accuracy computes feature importance by analyzing permuted out-of-bag samples and measuring the mean decrease in accuracy. In this study, the computing method described first was used.

According to Figure 3, the most effective first six features in distinguishing the two groups are, respectively: smoking, P-wave dispersion, P-wave amplitude, T-end P/(PQ*Age), Cornell product, and P-wave duration. Importance scores of other factors can be seen in Figure 1.

## 4. Discussion

AI enhances the quality of healthcare services by enabling more precise, rapid, and personalized diagnosis, treatment, and patient care in the medical field. The utilization of AI in analyzing vast medical data and predicting diseases improves early diagnosis and subsequently facilitates more effective management of treatment processes, leading to an enhancement in patients’ quality of life. Furthermore, AI plays a significant role in accelerating scientific discoveries and advancing the medical field in areas such as medical image analysis, genetic research, and drug development.

The current study investigated gender-specific ECG parameters of heart failure patients with preserved ejection fraction by using statistical analysis and AI methods. In this study, according to statistical analysis, electrocardiographic parameters, QRS duration, P-wave amplitude, P-wave duration, and P-wave dispersion were significantly higher in male patients with HFpEF than in female patients with HFpEF (*p* < 0.05). In addition, the rate of smoking was higher in men, while the rate of hypertension was higher in women (*p* < 0.05). However, according to AI, the most important parameters that distinguish male patients from female patients were as follows: smoking, P-wave dispersion, P-wave amplitude, T-end P/(PQ*Age), Cornell product, and P-wave duration. The RF model in this study showed very successful performance for distinguishing male patients with HFpEF from female patients with HFpEF. The average accuracy value for this algorithm was 0.847. The performance of the RF algorithm was also found to be successful in studies similar to this study [28]. After the RF algorithm, GBM was the method with the highest average performance evaluation metrics. After GBM, LR and SVM were effective algorithms, and their average performance evaluation criteria were fairly similar. On the other hand, kNN had the worst average performance of all the algorithms.

Although the performance of the kNN algorithm was reasonable, it had worse performance than the other algorithms used in this study. In general, kNN can sometimes return unsuccessful results due to the difficulty of neighborhood calculation in large datasets and high-dimensional data [29]. However, the dataset used in this study did not have these features. Therefore, it is thought that the low performance of kNN compared to other algorithms is due to the attributes in the dataset used in this study. Although this situation cannot be generalized, it can be said that RF and GBM algorithms were performed successfully on datasets similar to the dataset in this study.

In this study, feature importance scores were determined using AI algorithms. On the other hand, univariate statistical tests were applied to the variables considered in this study, and variables that showed statistical differences based on gender were identified. The variables that showed differences among them and the variables obtained using the feature importance function were largely similar. While univariate statistical methods measure significance by examining variables one by one, AI evaluates all variables together. Therefore, a variable or variables that may seem insignificant in univariate statistical analysis can turn out to be highly important when evaluated using AI. In conclusion, AI is preferred over statistical methods in some cases due to its advantages such as modeling complex relationships, working with large datasets, automatic feature engineering, and better generalization capabilities. However, the choice of which method to use may vary depending on the nature of the problem and the structure of the dataset.

The P-wave on the ECG represents the electrical depolarization of the atrium. In a healthy person, the P-wave is an ECG representation of electrical activity originating from the sinoatrial node (SA node), which is the depolarization of both the left and right atrium. Anatomical changes such as enlargement and fibrosis in the atrium can cause changes in the P-wave [30]. A previous study found a sensitivity and specificity of 98% and 64% for LVDD in patients with a P-wave dispersion >45 ms and who ruled out coronary artery disease (CAD) with a negative exercise test or coronary angiography (CAG) [31]. Another study found that P-wave dispersion and baseline troponin-I levels together were better than either parameter alone in predicting AF recurrence in patients with paroxysmal atrial fibrillation [32]. In another study, P-wave dispersion and duration were measured in 280 patients who underwent echocardiography for clinical indications (e.g., abnormal physical examination, hypertension, or suspected CAD or HF). It was determined that individuals with LVDD had higher P-wave dispersion and duration values than those without LVDD [33]. In another study involving a similar group of patients, P-wave duration >110 ms was found to be more sensitive (sensitivity 86%, specificity 86%) and P-wave duration >120 ms more specific (sensitivity 34% and specificity 100%) [34]. In a study of P-wave amplitude, in 204 LVDD patients without CAD or major cardiac pathology, there was 67% sensitivity and 60% specificity in detecting disease when the P-wave amplitude was above 0.102 mV [35]. In our study, according to descriptive statistics, P-wave dispersion, amplitude, and duration were significantly higher in male patients with HFpEF than in female patients with HFpEF. Similarly, according to AI, P-wave dispersion, amplitude, and duration were the most important electrocardiographic parameters that distinguish male patients with HFpEF from female patients with HFpEF.

On the ECG, the QRS complex represents electrical stimulation as it propagates through the ventricles and expresses ventricular depolarization [36]. Previous studies have found adverse cardiac outcomes in patients with reduced ejection fraction (HFrEF) heart failure associated with QRS duration [37,38]. In a study involving patients with HFpEF, QRS duration over 120 ms was found to be an important predictor of heart failure and hospitalization, but not associated with mortality [39]. However, in another study, prolonged QRS duration was found to be a predictor of poor prognosis in patients with HFpEF, especially QRS duration over 100 ms [40]. However, it remains unclear whether the duration of QRS has prognostic significance in patients with HFPEF. Although there are many studies on the duration of QRS in patients with HFrEF, there are few studies on patients with HFpEF. In addition, study groups often included patients with RBBB and LBBB. Patients with LBBB and HFpEF were not included in our study. According to the statistical analyses in our study, the duration of QRS was significantly higher in male patients with HFpEF than in female patients HFpEF. Similarly, according to AI, QRS duration was one of the important electrocardiographic parameters that distinguish male patients from female patients.

Smoking is one of the most important risk factors for cardiovascular diseases and lung cancer. Millions of people die every year due to smoking-related heart diseases and lung cancer. Although the relationship between cigarette smoking and the development of HFpEF is not clearly known, one study found that smoking is an independent risk factor for the development of heart failure with preserved ejection fraction [41]. In addition, other studies have found that smoking is an important predictor of death in patients with HFpEF [42,43]. In our study, according to the statistical analyses; smoking was significantly higher in male patients with HFpEF than in female patients with HFpEF. Similarly, according to AI, smoking was the most important parameter that distinguished male patients with HFpEF from female patients with HFpEF.

## 5. Limitations

This study has some limitations. First, the number of patients in this was study limited. Second, our data source included patients from only one geographic region of Turkey, which limits generalizability and requires validation in other populations. Third, we only compared gender-specific ECG parameters of male and female patients with HFpEF. We did not examine the healthy control group. Comparison between patients with HFpEF and the healthy control group could have provided greater insight. 

## 6. Conclusions

The proposed model serves as a valuable tool for physicians, facilitating diagnosis, treatment, and follow-up for distinguishing male patients with HFpEF from female patients with HFpEF. Analyzing readily accessible electrocardiographic parameters empowers medical professionals to make informed decisions and provide enhanced care to a wide range of individuals. In this study, we found that RF, one of the AI algorithms, helped identify electrocardiographic parameters for distinguishing male patients with HFpEF from female patients with HFpEF. P-wave duration, P-wave dispersion, QRS duration, and P-wave amplitude were the most effective ECG parameters. The ECG test, a widely available and cost-effective diagnostic tool, can be used to assess these parameters for distinguishing male patients with HFpEF from female patients with HFpEF. Moreover, we suggest employing AI in cardiology research to uncover more accurate risk factors for better risk assessment.

## Figures and Tables

**Table 1:** Descriptive statistics.

Demographics, Echocardiographics, and Electrocardiographics		Min	Max	Median	Mean ± SD	N	%
Group	Female patients with HFpEF					66	55.9
	Male patients with HFpEF					52	44.1
Cornell product (mm.ms)		700	2668	1186	1266.305 ± 355.064	118	100
Age		50	79	66.47	66.940 ± 7.863	118	100
T-end Q interval (ms)		260	680	420	422.457 ± 73.806	118	100
PR duration (ms)		112	214	160	158.991 ± 19.303	118	100
QRS duration (ms)		70	116	90	92.161 ± 9.905	118	100
OT (ms)		308	428	378	378.152 ± 23.851	118	100
Otc (ms)		367	453	410	410.737 ± 15.000	118	100
Heartrate		63	105	78	80.161 ± 9.524	118	100
P-wave amplitude (mV)		0.8	3	1.5	0.1566 ± 0.0423	118	100
P-wave duration (ms)		60	140	90	93.093 ± 18.056	118	100
P-wave dispersion (ms)		10	60	35	34.745 ± 13.311	118	100
T-end Q/(PQ*Age)		0.020	0.083	0.051	0.052 ± 0.012	118	100
T-end P/(PQ*Age)		0.010	0.065	0.033	0.034 ± 0.011	118	100
BMI		21.066	43.209	31.141	31.504 ± 4.488	118	100
HT	No					19	16.1
	Yes					99	83.9
Smoking	No					76	64.4
	Yes					42	35.6
DM	No					73	61.9
	Yes					45	38.1
LVESD (cm)		2.4	4.1	3.3	3.316 ± 0.350	118	100
LVEDD (cm)		4.1	5.6	4.9	4.439 ± 0.325	118	100
LA diameter (cm)		2.9	4.7	3.95	3.963 ± 0.233	118	100
PW thickness (cm)		0.8	10	1.1	1.168 ± 0.828	118	100
LVEF%		50	70	60	60.966 ± 3.307	118	100
IVS thickness (cm)		0.9	1.5	1.1	1.100 ± 0.127	118	100
E-wave (cm/s)		40	90	65	64.991 ± 9.572	118	100
A-wave (cm/s)		70	132	100	101.491 ± 10.166	118	100

Min: minimum; Max: maximum; SD: standard deviation; Otc: Ot correct interval; Cornell product: ((RaVL + SV3)*ORS duration); BMI: body mass index; HT: hypertension; DM: diabetes mellitus; LVESD: left ventricular end-systolic diameter; LVEDD: left ventricular end-diastolic diameter; LA diameter: left atrium diameter; PW: posterior wall; LVEF%: left ventricular ejection fraction; IVS: interventricular septum; mm: millimeter; cm: centimeter; ms: millisecond.

**Table 2:** Comparison of parameters (demographic, echocardiographic, and electrocardiographic) between female and male patients with HFpEF.

		Women	Men	
		Mean ± SD	Mean ± SD	p
Cornell product (mm.ms)		1265.818 ± 351.614	1266.923 ± 362.834	0.721
Age		66.757 ± 7.625	67.173 ± 8.224	0.777
T-end Q interval (ms)		426.969 ± 66.354	416.730 ± 82.615	0.457
PR duration (ms)		158.060 ± 17.861	160.173 ± 21.111	0.718
QRS duration (ms)		89.833 ± 8.477	95.115 ± 10.844	0.011 *
OT (ms)		378.424 ± 21.481	377.807 ± 26.772	0.893
Otc (ms)		410.697 ± 14.591	410.788 ± 15.647	0.974
Heartrate		79.363 ± 9.189	81.173 ± 9.930	0.301
P-wave amplitude (mV)		0.1465 ± 0.0414	0.1696 ± 0.0402	0.001 **
P-wave duration (ms)		87.045 ± 16.171	100.769 ± 17.528	<0.001 **
P-wave dispersion (ms)		29.697 ± 11.150	41.153 ± 13.158	<0.001 **
T-end Q/(PQ*Age)		0.053 ± 0.011	0.051 ± 0.014	0.513
T-end P/(PQ*Age)		0.035 ± 0.009	0.033 ± 0.012	0.375
BMI		32.149 ± 4.250	30.685 ± 4.686	0.106
		**N (%)**	**N (%)**	
HT	No	6 (9.1)	13 (25)	0.020 *
	Yes	60 (90.9)	39 (75)	
Smoking	No	57 (86.4)	19 (36.5)	<0.001 **
	Yes	9 (13.6)	33 (63.5)	
DM	No	36 (54.5)	37 (71.2)	0.065
	Yes	30 (45.5)	15 (28.8)	
		**Mean** ± **SD**	**Mean** ± **SD**	
LVESD (cm)		3.290 ± 0.344	3.348 ± 0.357	0.427
LVEDD (cm)		4.898 ± 0.342	4.990 ± 0.297	0.109
LA diameter (cm)		3.968 ± 0.255	3.955 ± 0.204	0.580
PW thickness (cm)		1.234 ± 1.101	1.084 ± 0.117	0.305
LVEF%		61.045 ± 3.135	60.865 ± 3.542	0.720
IVS thickness (cm)		1.101 ± 0.123	1.100 ± 0.134	0.690
E-wave (cm/s)		66.560 ± 9.741	63.000 ± 9.057	0.043 *
A-wave (cm/s)		103.257 ± 11.434	99.250 ± 7.831	0.148

SD: standard deviation; *: difference is significant at the 0.05 level (two-tailed); **: difference is significant at the 0.01 level (two-tailed).

**Table 3:** Average performance evaluation metrics with 95% confidence intervals (CIs) for the classification algorithms.

Algorithm	GBM (95% CI)	kNN (95% CI)	LR (95% CI)	RF (95% CI)	SVM (95% CI)
Accuracy	0.806 (0.761, 0.851)	0.486 (0.396, 0.576)	0.764 (0.712, 0.817)	0.847 (0.807, 0.887)	0.750 (0.692, 0.808)
Precision	0.874 (0.835, 0.913)	0.442 (0.343, 0.541)	0.750 (0.696, 0.804)	0.867 (0.827, 0.907)	0.731 (0.678, 0.784)
Recall	0.672 (0.606, 0.739)	0.502 (0.426, 0.578)	0.678 (0.612, 0.744)	0.792 (0.742, 0.842)	0.678 (0.611, 0.745)
*F1* score	0.756 (0.702, 0.810)	0.439 (0.338, 0.541)	0.708 (0.648, 0.768)	0.826 (0.785, 0.868)	0.697 (0.634, 0.761)
Kappa score	0.596 (0.529, 0.663)	0.006 (−0.116, 0.128)	0.494 (0.414, 0.575)	0.681 (0.616, 0.746)	0.468 (0.373, 0.561)
AUC	0.827 (0.785, 0.869)	0.490 (0.405, 0.575)	0.875 (0.836, 0.914)	0.902 (0.869, 0.934)	0.808 (0.763, 0.851)

## Data Availability

Data are available in Supplementary Material Table S1.

## Supplementary Materials

The following supporting information can be downloaded at: https://www.mdpi.com/article/10.3390/diagnostics13203221/s1, Table S1: Dataset.

## References

[1] Shirazi L.F., Bissett J., Romeo F., Mehta J.L. (2017). Role of inflammation in heart failure. Curr. Atheroscler. Rep..

[2] Steinberg B.A., Zhao X., Heidenreich P.A., Peterson E.D., Bhatt D.L., Cannon C.P., Fonarow G.C. (2012). Get With the Guidelines Scientific Advisory Committee and Investigators. Trends in patients hospitalized with heart failure and preserved left ventricular ejection fraction: Prevalence, therapies, and outcomes. Circulation.

[3] Redfield M.M., Jacobsen S.J., Burnett J.C., Mahoney D.W., Bailey K.R., Rodeheffer R.J. (2003). Burden of systolic and diastolic ventricular dysfunction in the community: Appreciating the scope of the heart failure epidemic. JAMA.

[4] Savarese G., Becher P.M., Lund L.H., Seferovic P., Rosano G.M., Coats A.J. (2023). Global burden of heart failure: A comprehensive and updated review of epidemiology. Cardiovasc. Res..

[5] McDonagh T.A., Metra M., Adamo M., Gardner R.S., Baumbach A., Böhm M., Kathrine Skibelund A. (2021). 2021 ESC Guidelines for the diagnosis and treatment of acute and chronic heart failure: Developed by the Task Force for the diagnosis and treatment of acute and chronic heart failure of the European Society of Cardiology (ESC) With the special contribution of the Heart Failure Association (HFA) of the ESC. Eur. Heart J..

[6] Nagueh S.F., Smiseth O.A., Appleton C.P., Byrd B.F., Dokainish H., Edvardsen T., Flachskampf F.A., Gillebert T.C., Klein A.L., Lancellotti P. (2016). Recommendations for the evaluation of left ventricular diastolic function by echocardiography: An update from the American Society of Echocardiography and the European Association of Cardiovascular Imaging. J. Am. Soc. Echocardiogr..

[7] Pieske B., Tschöpe C., De Boer R.A., Fraser A.G., Anker S.D., Donal E., Edelmann F., Fu M., Guazzi M., Lam C.S. (2019). How to diagnose heart failure with preserved ejection fraction: The HFA-PEFF diagnostic algorithm: A consensus recommendation from the Heart Failure Association (HFA) of the European Society of Cardiology (ESC). Eur. Heart J..

[8] Magnani J.W., Wang N., Nelson K.P., Connelly S., Deo R., Rodondi N., Schelbert E.B., Garcia M.E., Phillips C.L., Shlipak M.G. (2013). The electrocardiographic PR interval and adverse outcomes in older adults: The health, aging and body composition study. Circ. Arrhythm. Electrophysiol..

[9] Kataoka H., Madias J.E. (2011). Changes in the amplitude of electrocardiogram QRS complexes during follow-up of heart failure patients. J. Electrocardiol..

[10] Dhingra R., Pencina M.J., Wang T.J., Nam B.H., Benjamin E.J., Levy D., Larson M.G., Kannel W.B., D’Agostino Sr R.B., Vasan R.S. (2006). Electrocardiographic QRS duration and the risk of congestive heart failure: The Framingham heart study. Hypertension.

[11] Coronel R., Wilders R., Verkerk A.O., Wiegerinck R.F., Benoist D., Bernus O. (2013). Electrophysiological changes in heart failure and their implications for arrhythmogenesis. Biochim. Biophys. Acta.

[12] Houghton A.R., Sparrow N.J., Toms E., Cowley A.J. (1997). Should general practitioners use the electrocardiogram to select patients with suspected heart failure for echocardiography?. Int. J. Cardiol..

[13] Daamen M.A., Brunner-la Rocca H.P., Tan F.E., Hamers J.P., Schols J.M. (2017). Clinical diagnosis of heart failure in nursing home residents based on history, physical exam, BNP and ECG: Is it reliable?. Eur. Geriatr. Med..

[14] Nikolaidou T., Samuel N.A., Marincowitz C., Fox D.J., Cleland J.G.F., Clark A.L. (2020). Electrocardiographic characteristics in patients with heart failure and normal ejection fraction: A systematic review and meta-analysis. Ann. Noninvasive Electrocardiol..

[15] Paksoy N., Yagin F.H. (2022). Artificial intelligence-based colon cancer prediction by identifying genomic biomarkers. Med. Rec..

[16] Koulaouzidis G., Jadczyk T., Iakovidis D.K., Koulaouzidis A., Bisnaire M., Charisopoulou D. (2022). Artificial intelligence in cardiology—A narrative review of current status. J. Clin. Med..

[17] Lang R.M., Badano L.P., Mor-Avi V., Afilalo J., Armstrong A., Ernande L., Flachskampf F.A., Foster E., Goldstein S.A., Kuznetsova T. (2015). Recommendations for cardiac chamber quantification by echocardiography in adults: An update from the American Society of Echocardiography and the European Association of Cardiovascular Imaging. Eur. Heart J. Cardiovasc. Imaging.

[18] Natekin A., Knoll A. (2013). Gradient boosting machines, a tutorial. Front. Neurorobot..

[19] Zhang Z. (2016). Introduction to machine learning: K-nearest neighbors. Ann. Transl. Med..

[20] Dangeti P. (2017). Statistics for Machine Learning.

[21] Padilla R., Netto S.L., da Silva E.A.B. A Survey on Performance Metrics for Object-Detection Algorithms. Proceedings of the International Conference on Systems, Signals and Image Processing (IWSSIP).

[22] Cohen J. (1960). A coefficient of agreement for nominal scales. Educ. Psychol. Meas..

[23] Artstein R., Poesio M. (2008). Inter-coder agreement for computational linguistics. Comput. Linguist..

[24] Powers D.M.W. (2011). Evaluation: From Precision, recall and F-measure to ROC, informedness, markedness & correlation. J. Mach. Learn. Technol..

[25] Bohara U. (2020). Student Retention Analysis. Proceedings of the Third Annual Great Lakes Data Science Sympoisum.

[26] Liang J., Liu Q., Nie N., Zeng B., Zhang Z. (2019). An Improved Algorithm based on KNN and Random Forest. Proceedings of the 3rd International Conference on Computer Science and Application Engineering (CSAE ‘19).

[27] Gupta D., Sundaram S., Khanna A., Hassanien A.E., De Albuquerque V.H.C. (2018). Improved diagnosis of Parkinson’s disease using optimized crow search algorithm. Comput. Electr. Eng..

[28] Deo R.C. (2015). Machine learning in medicine. Circulation.

[29] Shokrzade A., Ramezani M., Tab F.A., Mohammad M.A. (2021). A novel extreme learning machine based kNN classification method for dealing with big data. Expert Syst. Appl..

[30] Baranchuk A., Bayés de Luna A. (2015). The P-wave morphology: What does it tell us?. Herzschrittmacherther. Elektrophysiol..

[31] Taha T., Sayed K., Saad M., Samir M. (2016). How accurate can electrocardiogram predict left ventricular diastolic dysfunction?. Egypt. Heart J..

[32] Ocak M., Tascanov B.T. (2021). Clinical value of the combined use of P-wave dispersion and troponin values to predict atrial fibrillation recurrence in patients with paroxysmal atrial fibrillation. Rev. Port. Cardiol..

[33] Tsai W.C., Lee K.T., Chu C.S., Lin T.H., Hsu P.C., Su H.M., Voon W.C., Lai W.T., Sheu S.H., Wu M.T. (2013). Significant correlation of P-wave parameters with left atrial volume index and left ventricular diastolic function. Am. J. Med. Sci..

[34] Sumita Y., Nakatani S., Murakami I., Taniguchi M. (2020). Significance of left atrial overload by electrocardiogram in the assessment of left ventricular diastolic dysfunction. J. Echocardiogr..

[35] Hayıroğlu M.İ., Tufan Ç., Vedat Ç., Süha A., Şahhan K., Nurgül K., Mehmet U., Lütfullah O.A. (2021). A simple formula to predict echocardiographic diastolic dysfunction—Electrocardiographic diastolic index. Herz.

[36] Hampton J., Hampton J. (2019). The ECG Made Easy.

[37] Mentz R.J., Greiner M.A., DeVore A.D., Dunlay S.M., Choudhary G., Ahmad T., Khazanie P., Randolph T.C., Griswold M.E., Eapen Z.J. (2015). Ventricular conduction and long-term heart failure outcomes and mortality in African Americans: Insights from the Jackson heart study. Circ. Heart Fail..

[38] Silvet H., Amin J., Padmanabhan S., Pai R.G. (2001). Prognostic implications of increased QRS duration in patients with moderate and severe left ventricular systolic dysfunction. Am. J. Cardiol..

[39] Reinier K., Narayanan K., Uy-Evanado A., Teodorescu C., Chugh H., Mack W.J., Gunson K., Jui J., Chugh S.S. (2015). Electrocardiographic markers and left ventricular ejection fraction have cumulative effects on Risk of Sudden Cardiac Death. JACC Clin. Electrophysiol..

[40] Lund L.H., Jurga J., Edner M., Benson L., Dahlström U., Linde C., Alehagen U. (2013). Prevalence, correlates, and prognostic significance of QRS prolongation in heart failure with reduced and preserved ejection fraction. Eur. Heart J..

[41] Ho J.E., Lyass A., Lee D.S., Vasan R.S., Kannel W.B., Larson M.G., Levy D. (2013). Predictors of new-onset heart failure: Differences in preserved versus reduced ejection fraction. Circ. Heart Fail..

[42] Yap J., Sim D., Lim C.P., Chia S.Y., Go Y.Y., Jaufeerally F.R., Sim L.L., Liew R., Ching C.K. (2015). Predictors of two-year mortality in Asian patients with heart failure and preserved ejection fraction. Int. J. Cardiol..

[43] Pocock S.J., Ariti C.A., McMurray J.J., Maggioni A., Køber L., Squire I.B., Swedberg K., Dobson J., Poppe K.K., Whalley G.A. (2013). Predicting survival in heart failure: A risk score based on 39 372 patients from 30 studies. Eur. Heart J..

